# Effect of carbohydrate-protein supplement timing on acute exercise-induced muscle damage

**DOI:** 10.1186/1550-2783-5-5

**Published:** 2008-02-19

**Authors:** James P White, Jacob M Wilson, Krista G Austin, Beau K Greer, Noah St John, Lynn B Panton

**Affiliations:** 1 Department of Exercise Science, University of South Carolina Columbia, SC 29101, USA; 2 Department of Nutrition, Food and Exercise Sciences, Florida State University, Tallahassee, FL 32306, USA

## Abstract

**Purpose:**

To determine if timing of a supplement would have an effect on muscle damage, function and soreness.

**Methods:**

Twenty-seven untrained men (21 ± 3 yrs) were given a supplement before or after exercise. Subjects were randomly assigned to a pre exercise (n = 9), received carbohydrate/protein drink before exercise and placebo after, a post exercise (n = 9), received placebo before exercise and carbohydrate/protein drink after, or a control group (n = 9), received placebo before and after exercise. Subjects performed 50 eccentric quadriceps contractions on an isokinetic dynamometer. Tests for creatine kinase (CK), maximal voluntary contraction (MVC) and muscle soreness were recorded before exercise and at six, 24, 48, 72, and 96 h post exercise. Repeated measures ANOVA were used to analyze data.

**Results:**

There were no group by time interactions however, CK significantly increased for all groups when compared to pre exercise (101 ± 43 U/L) reaching a peak at 48 h (661 ± 1178 U/L). MVC was significantly reduced at 24 h by 31.4 ± 14.0%. Muscle soreness was also significantly increased from pre exercise peaking at 48 h.

**Conclusion:**

Eccentric exercise caused significant muscle damage, loss of strength, and soreness; however timing of ingestion of carbohydrate/protein supplement had no effect.

## Background

Both prolonged endurance [1,2] and short bouts of high intensity [3] exercise are associated with elevated muscle tissue damage; a process characterized by disruption of the sarcoplasmic reticulum and sarcomeric Z-line proteins [4]. Currently it is thought that mechanically induced trauma from physical exertion triggers a metabolic cascade of events characterized by progressive increases in microscopic indicators of muscle damage [5,6]. Some indicators of muscle damage include increased levels of creatine kinase (CK), lactate dehydrogenase (LDH) and muscular soreness [7]. Increased indices of muscle damage are associated with decreased performance. Nosaka et al. [8] found significant correlations between reduction in maximum voluntary contraction (MVC) and changes in relaxed and flexed elbow joint angles, range of motion, arm circumference, peak palpation and extension muscle soreness, and peak CK activity. Numerous athletic events (e.g. sports requiring multiple periods, weight lifting, triathlons) and training procedures (double sessions) require participants to exert repeated efforts while maintaining peak performance throughout. Exercise nutritionists have therefore sought to investigate possible interventions which can lower acute indices of muscle damage [1,2].

Of particular interest has been the findings that amino acids, [9] amino acid based metabolites [10], whole proteins [1,2], or their combination with carbohydrates have been demonstrated to decrease markers of muscle damage under a variety of conditions [1-3,9]. As an example, Coombes et al. [9] administered 12 grams of branched chain amino acids (BCAAs) for two weeks prior to a 120 minute, moderate intensity cycle ergometer test, with an additional 20 grams administered immediately before and after the test. The supplemented group had lower CK and LDH levels following the exercise bout than the placebo condition. In a more recent study Shimomura et al. [11] administered 5 grams of BCAAs or a placebo to men and women (21–24 years of age) prior to resistance exercise and found that muscle soreness was lower in both sexes after BCAA ingestion compared to a placebo. In addition, results from Bird et al. [12,13] suggest that carbohydrates act synergistically with essential amino acids (EAAs) following resistance exercise to lower 3-methylhistidine levels, and increase lean body mass. 3-methylhistidine is an amino acid mainly concentrated in skeletal muscle that is used as an indicator of actomyosin protein breakdown [5] and muscle fiber damage [5,14].

Amino acids are thought to exert their protective effects through a conglomeration of direct and indirect mechanisms. Directly, amino acids may depress pathways responsible for Z-Line disruption during the metabolic cascade triggered by mechanical trauma, [4,15] while indirect mechanisms relate to formation of specific amino acid derived metabolites, such as Beta-Hydroxy-Beta-Methylbutyrate, shown in studies to lower indices of muscle damage [10].

As with free form amino acids, a combination of whole proteins/carbohydrates ingested before, after, or during endurance exercise has resulted in lowered post exercise muscle soreness [16], and plasma concentrations of both myoglobin [2] and CK [1,2]. In contrast, a study which administered a milk protein and carbohydrate solution immediately following resistance training found no differences between the supplemented and placebo conditions on muscle soreness or maximal voluntary strength [3]. However, there was a non significant trend for CK levels to be lower in the group administered the milk and carbohydrate solution on the day of eccentric resistance exercise as compared to the placebo condition. Lower levels of 3-methylhistidine were also found on the day of the resistance training bout [3]. The lower clarity of these results may be attributed to the methodology utilized. Specifically in both studies participants performed exhaustive cycling bouts to deplete intramuscular glycogen on the day prior to the resistance training bout, leading to increased levels of CK prior to the actual resistance training bout.

Currently there is little research measuring indices of muscle damage when a whole protein supplement is administered immediately prior or immediately following resistance exercise, and the research that has been conducted has been difficult to interpret. There is also little research on the overall timing of an intact protein combined with a carbohydrate supplement on indices of muscle damage. Therefore the purpose of this study was to determine the effects of a carbohydrate and protein supplement ingested immediately prior to eccentric resistance exercise as compared to immediately following an eccentric resistance exercise bout on muscle damage measured by serum CK, MVC, and muscle soreness.

## Methods

### Subjects

Twenty-seven healthy college-aged men between 18–25 years of age were recruited. Subjects were sedentary and had not participated in a resistance or an aerobic exercise program for at least six months. Each subject signed an informed consent approved by the University Institutional Review Board before participating in the study.

### Experimental design

On the first visit subjects reported to the laboratory after an overnight 12-hour fast. Body composition was estimated using the sum of three skinfolds (chest, abdomen, thigh) following the procedures outlined by Jackson and Pollock [17]. Baseline muscle soreness was evaluated using the visual analogue scale (VAS) which has been utilized as a valid indicator of pain in several studies [3,8,11,18] has correlated with other indices of muscle damage including MVC [8], and CK [8], and has obtained reported reliability scores as high as *r *= 0.97 for assessing soreness [19]. A baseline blood draw was taken to measure serum CK levels. The first drink, either placebo or the carbohydrate/protein supplement, was given after the above tests were completed. The subjects had two minutes to consume the beverage. Following ingestion of the drink, the last test of the baseline measurements was completed. This included an isometric strength test using a maximal voluntary contraction (MVC) of the quadriceps [20]. The subjects performed the exercise bout roughly 15 minutes after they consumed the drink. Immediately after the completion of the exercise bout, the subjects consumed the second drink. Again, two minutes were given to consume the drink. Subjects repeated all the measurements at six, 24, 48, 72, and 96 hours after exercise. Measurements up to 96 hours were taken due to the general delayed response of indicators of muscle damage, which typically peak at 72–96 hours following eccentric exercise [3]. Three hours and thirty minutes (210 min) after the completion of the exercise bout, the subjects were given a meal consisting of Equate^© ^(6 grams fat, 40 grams carbohydrate, 9 grams protein). This meal was given to taper hunger levels while ensuring all subjects consumed an equal amount of calories and macronutrient content. Subjects had two minutes to consume the liquid meal. After the meal, the subjects were able to leave the laboratory if needed before the six-hour measurements were taken. No food was consumed between the Equate^© ^meal and the six-hour measurements. At 24, 48, 72, and 96 hours after the exercise bout all measurements were taken after 12 hours of overnight fasting. All subjects were instructed to consume their normal diet as well as keep daily activity to a minimum. This attempted to control for unusual nutritional habits or physical activity. In addition, subjects were told to refrain from taking any vitamin supplementation or anti-inflammatory medications to prevent any further nutritional or drug-related protection against the exercise-induced muscle damage. These restrictions were enforced 48 hours before and during the testing period.

### Resistance exercise protocol

The resistance exercise protocol was completed on an isokinetic dynamometer (Biodex™, Shirley, New York). The subject's upper and lower body were restricted with shoulder and leg straps to help isolate the exercising leg. Subjects completed ten sub-maximal concentric/concentric contractions of the quadriceps and hamstrings of the dominant leg for a warm up. They were then tested for the MVC one minute after the completion of the warm up. All testing for MVC was performed with the subject's knee joint placed at a 90° angle. For each measurement period the subject performed three maximal isometric contractions of the knee extensors for five seconds separated by a ten second rest. The three MVC trials were averaged together to give the score for that particular measurement period. Intraclass correlation coefficients have been reported for knee extension isometric peak torque values ranging from 0.82 to 0.89 in healthy and patient populations [21]. A three-minute rest period was given before the subject completed 50 maximal isokinetic eccentric quadriceps contractions on the same leg that performed the warm up and MVC test. The exercise bout was divided into five sets of ten repetitions with a minute rest interval between sets. For all contractions the Biodex™ was set through a range of motion of 1.75 rad (100°). Each contraction was performed at an angular velocity of 1.05 rad/s (60°/s).

### Creatine kinase

Five mL of blood was taken from an antecubital vein using sterile venipuncture techniques. Blood was collected in untreated vacutainers and centrifuged for 10 minutes. Samples were separated and stored at -80°C until analysis. Serum CK activity was measured in duplicate using an enzymatic assay kit (StanBio Laboratories; Borne, Texas) after all samples had been collected. After each set of samples was collected, a standard amount of CK was frozen with the samples to measure the activity changes due to freezing. Each sample in that set was corrected against an activity change of the frozen CK standard. Samples were frozen for no longer than five days before being analyzed. The coefficient of variation for CK determination was within 4%.

### Experimental drink

All subjects were randomly assigned to one of three groups. The first group drank the carbohydrate/protein drink 15 minutes before the exercise and the placebo drink after the exercise. The second group consumed the placebo drink 15 minutes before exercise and the carbohydrate/protein drink second. The third group consumed the placebo drink first followed with another placebo drink after the exercise. The carbohydrate/protein drink consisted of 23 grams of whey protein and 75 grams of carbohydrate (392 kcals) mixed with 300 ml of water. The amino acid array consisted of 400 mg of histidine, 1520 mg of isoleucine, 2470 mg of leucine, 2120 mg of lysine, 440 mg of methionine, 670 mg of phenylalanine, 1720 mg of threonine, 1440 mg of valine, 480 mg of arginine, 440 mg of cystine, 590 mg of tyrosine, 1540 mg of praline, 3870 mg of glutamine, 1240 mg of serine, 530 mg of glycine, 1380 mg of alanine, and 2490 mg of aspartic acid. The carbohydrate in the supplement was made up of dextrose, maltodexrin and fructose. The placebo solution was composed of water and an artificial sweetener (Splenda^©^, Ft. Washington, PA) to improve palatability. To make certain the experimenters and subjects did not know which group they were working with or were in, aluminum foil was wrapped around each container to hide color and content of the drink.

### Statistical analysis

A one-way analysis of variance (ANOVA) was used to compare groups on baseline values. A 3 × 6 (group × time) repeated ANOVA was used to test for significance between CK values, MVC values, and VAS scores. A 3 × 5 (group × time) repeated ANOVA was used to test for significance when MVC values were normalized to a percentage. Since the Mauchly's Test of Sphericity was violated, the Greenhouse-Geisser test was used to measure significance. A Tukey HSD post hoc test was used to locate significance between time points if there was a main effect of time. All significance was accepted at *p *= 0.05. Values in tables are presented as means ± standard deviations and values in figures are presented as means ± standard errors. All statistical procedures were carried out on SPSS version 13.0.

## Results

### Subject characteristics

There were no differences among groups for age, body weight, height, percent body fat or lean mass (Table 1). All subjects verbally confirmed they had been sedentary for at least six months and were not taking any nutritional supplements prior to the study.

**Table 1:** Subject characteristics (N = 27).

Group	Age(yr)	Height(m)	Weight(kg)	Lean Mass (kg)	% Body Fat
Control(n = 9)	20.6 ± 02.1	1.79 ± 0.09	78.1 ± 11.7	68.2 ± 09.1	12.4 ± 05.2
Pre (n = 9)	21.6 ± 02.4	1.80 ± 0.06	89.5 ± 23.4	73.3 ± 09.0	15.7 ± 10.9
Post (n = 9)	21.1 ± 02.2	1.77 ± 0.10	81.3 ± 13.7	68.1 ± 10.0	15.6 ± 09.1

Values are means ± standard deviation. Pre, received supplement before exercise and placebo after; Post, received supplement after exercise and placebo before exercise; Control, received placebo before and after exercise.

### Strength

**Figure 1:**
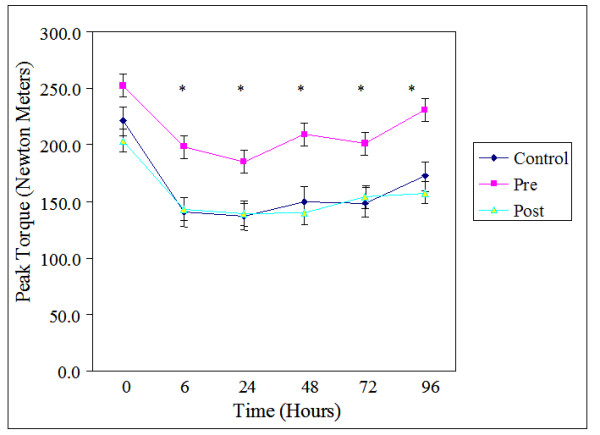
**Peak torque of maximal voluntary contraction (MVC) across the 96 hours.** Values are means ± standard errors, * *p *< 0.05, significantly different from 0 time point, there were no differences among groups. Pre, received supplement before exercise and placebo after; Post, received supplement after exercise and placebo before exercise; Control, received placebo before and after exercise.

**Figure 2:**
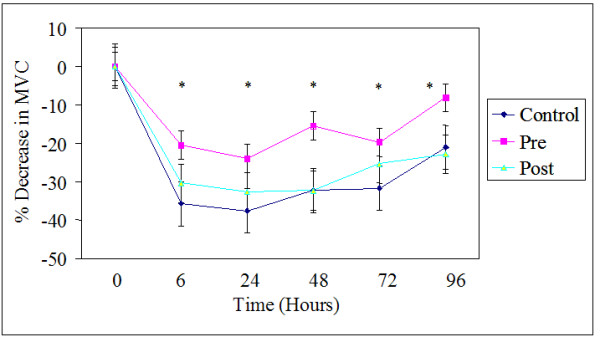
**Percent decrease of maximal voluntary contraction (MVC) across the 96 hours.** Values are means ± standard errors. Values for % decrease in MVC = ((Pre – time point)/Pre) × 100. * *p *< 0.05, significantly different from 0 time point, there were no differences among groups. Pre, received supplement before exercise and placebo after; Post, received supplement after exercise and placebo before exercise; Control, received placebo before and after exercise.

Baseline maximal voluntary contraction (MVC) scores were similar for all three groups (Table 2). There was no group by time interaction for MVC values, however there was a significant time effect (F(5,90) = 28.579, *p *≤ 0.05, ES = 0.61). After the exercise bout, MVC scores were significantly lower for all time points compared to the pre exercise values (Figure 1). After the initial decrease in strength at 6, the 24, 48, and 72 h time points were not different from each other. When the MVC scores were converted to percent decrease from the pre exercise values, the results were similar (F(4,72) = 7.095, *p *≤ 0.05, ES = 0.28; Figure 2). All time points were significantly lower from pre exercise values. The supplement or timing of ingestion had no effect on MVC scores over time.

**Table 2:** Values for CK, MVC, and VAS for the three supplement groups (N = 27).

Variables		0	6 hrs	24 hrs	48 hrs	72 hrs	96 hrs
CK	Control	94 ± 63	153 ± 134	245 ± 178	1084 ± 1709*	1428 ± 2208*	1181 ± 1540*
(U/L)	Pre	113 ± 031	184 ± 098	271 ± 273	0620 ± 1098*	1050 ± 2189*	0873 ± 1513*
(n = 9)	Post	97 ± 28	139 ± 057	182 ± 059	280 ± 115*	424 ± 286*	634 ± 787*
MVC	Control	221 ± 37	140 ± 21*	137 ± 31*	150 ± 053*	149 ± 038*	172 ± 051*
(NM)	Pre	252 ± 66	198 ± 43*	185 ± 26*	209 ± 047*	201 ± 049*	231 ± 059*
(n = 9)	Post	204 ± 29	143 ± 040*	139 ± 046*	140 ± 047*	154 ± 047*	157 ± 039*
VAS	Control	0.11 ± 0.17	1.19 ± 1.45*	1.84 ± 1.15*	3.59 ± 1.56*	2.60 ± 1.37*	1.09 ± 1.03*
(cm)	Pre	0.03 ± 0.07	0.66 ± 0.60*	2.22 ± 1.28*	3.44 ± 1.82*	2.54 ± 1.56*	1.48 ± 1.48*
(n = 9)	Post	0.02 ± 0.04	0.73 ± 0.96*	1.54 ± 1.33*	2.12 ± 1.58*	1.22 ± 1.03*	0.63 ± 0.51*

Values are means ± standard deviation.

* *p *< 0.05, significantly different from 0 time point

Pre, received supplement before exercise and placebo after; Post, received supplement after exercise and placebo before exercise; Control, received placebo before and after exercise; CK, creatine kinase; MVC, maximal voluntary contraction; NM, newton meters; VAS, visual analogue scale.

### Muscle damage

**Figure 3:**
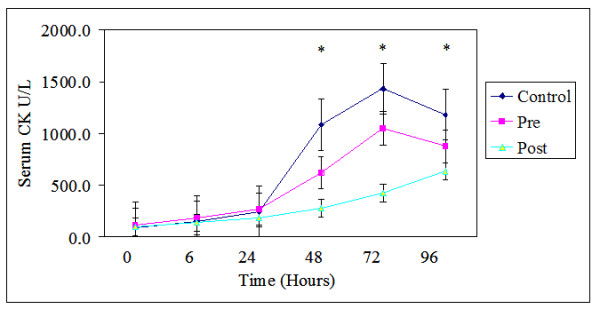
**Serum creatine kinase (CK) Concentration across the 96 hours.** Values are means ± standard errors. * *p *< 0.05, significantly different from 0 time point, there were no differences among groups. Pre, received supplement before exercise and placebo after; Post, received supplement after exercise and placebo before exercise; Control, received placebo before and after exercise.

There were no differences in serum CK values among the three groups at baseline. There was no group by time interaction after the exercise bout. However, there was a significant time effect (F(1.123,20.205) = 5.766, *p *≤ 0.05, ES = 0.24). Creatine kinase values significantly increased across time for all three groups from baseline (Table 2; Figure 3). Peak CK concentration was at 48 h post exercise. The supplement or timing of ingestion had no effect on serum CK at any time points.

### Muscle soreness

**Figure 4:**
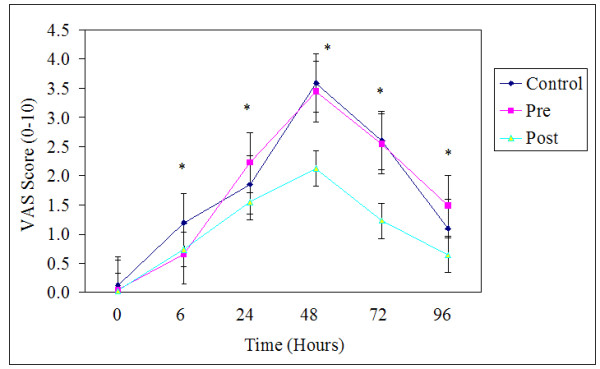
**Visual analogue scale (VAS) across the 96 hours.** Values are means ± standard errors. * *p *< 0.05, significantly different from 0 time point, there were no differences among groups. Pre, received supplement before exercise and placebo after; Post, received supplement after exercise and placebo before exercise; Control, received placebo before and after exercise

Pre exercise values were not different among groups for muscle soreness. There was no group by time interaction, but there was a main effect of time. Muscle soreness significantly increased above baseline levels for all groups at all time points (F(2.914,52.460) = 24.473, *p *≤ 0.05, ES = 0.58; Figure 4). Peak soreness occurred at 48 h post exercise for all groups. Once again, the supplement or timing of ingestion had no effect on soreness scores.

## Discussion

This study was designed to determine whether the response of muscle tissue damage to a carbohydrate and protein supplement differed if ingested immediately prior to eccentric resistance exercise as compared to immediately following eccentric resistance exercise. Muscle damage was chemically inferred through plasma CK levels, functionally through isometric MVC of the knee extensors, and muscle soreness through the use of a VAS. This study found that there were no differences in muscle damage, function, or soreness between the timing of the carbohydrate and protein supplement.

### Creatine kinase

Creatine kinase values and muscular soreness increased across time for all three groups from baseline (Table 2). However no significant differences were found among groups at any point in time. These results disagreed with a number of endurance training studies which found that BCAAs [9], or whole protein/carbohydrate ingestion lowered plasma CK [1,2], myoglobin [2], muscle soreness [16], and maximum voluntary strength [18]. However, these findings partially agreed with Wojcik and colleagues [3] who found that consumption of a carbohydrate and whole protein solution 6 hours following resistance exercise only had a trend (*p *< 0.08) to decrease plasma CK levels at early time points after exercise.

One possible explanation for discrepancies seen may be related to the dose of BCAAs contained in the protein administered in the present study. Specifically we gave a 23 gram bolus of protein containing approximately 5 grams of BCAAs. However, in the Coombes et al. [9] investigation previously discussed, participants were administered a large 12 gram dose of BCAAs each day for two weeks prior to the exercise bout, followed by a 20 gram bolus immediately prior to and following exercise. Under these conditions, it is more likely that the BCAAs exerted both direct (extra cellular signaling) and indirect (conversion to Beta-Hydroxy-Beta-Methylbutyrate) effects on muscle tissue damage. However, this explanation is not supported by Ohtani et al. [18] who found that a small free form amino acid mixture (5.6 grams) ingested directly before and after eccentric exercise in both humans and animals lowered CK levels.

A second explanation may be related to the form of ingested amino acids. We administered amino acids via a whole protein source, while Coombes et al. [9] and Ohtani et al. [18] administered free form amino acids. It has been well established that the digestion rate of a protein or amino acid supplement can affect measures of protein balance [22]. While we did not measure protein balance, digestion rate may also affect indices of muscle damage. Further, immediate ingestion of whole proteins prior to exercise may not have provided a temporally adequate environment for a large enough increase in extra cellular amino acid concentrations during resistance exercise to exert effects on muscle tissue damage as compared to after resistance exercise protein ingestion. This suggestion appears to be supported by endurance training studies which demonstrate more consistent decreases in markers of muscle damage following ingestion of intact proteins before or during exercise [1,2,16]. Specifically, endurance activities are more prolonged than resistance training bouts, which may allow adequate time for whole proteins to digest and exert their protective effects on muscle tissue. Future research will need to elucidate if this relationship exists.

A third possibility is related to the dependent measure of CK itself. According to the raw data in Table 2 the pre condition had a nearly 80% lower concentration of CK at 48 hours than the control. However, as with past studies, [8,23,24] the CK response in the current experiment was highly variable and these results did not reach significance even when separate analyses were conducted to normalize the data, or remove possible outliers. The variable response appears to be related to the size of CK [24]. Specifically larger proteins such as CK are first taken up by lymphatic vessels, where their release into blood plasma is delayed by the relatively slow fluid movement of the system. Flow in the lymphatic vessels can be facilitated by the physical movement of the subject. Therefore the variability of the CK response could be attributed to the variability in movement of human participants, even when activity in the following days was minimized.

### Isometric maximal voluntary contraction

Data indicate significant correlations between MVC, plasma, functional, and reported indices of muscle damage, suggesting that MVC is a valid indirect measure of muscle damage [8]. In the present study, MVC was lower at 6, 24, 48, and 72 h time points for all three groups. While there were no significant differences between groups at any time, there was a somewhat lower decrease in MVC in the pre carbohydrate/protein group, relative to the post carbohydrate/protein and placebo groups (Figure 2, 3). Wojcik et al [3] also found no differences between groups in depression of MVC when a whole protein was administered following exercise. However, these findings disagreed with a recent study conducted by Ohtani and colleagues [18], who found significantly lower depression of both isometric and eccentric strength when free form amino acids were ingested following exercise. As before, it is possible that the digestion rate of intact proteins, relative to free form amino acids at least partially explains discrepant results.

### Muscular soreness

While the exact mechanisms of muscular soreness have not fully been elucidated, it is generally accepted that soreness is associated with the cascade of events which follow initial mechanically induced perturbations [25]. Following initial trauma an increase in intracellular Ca^++ ^levels stimulates degradative pathways responsible for further Z-line disruption [4]. The compromised state of the myofiber triggers an inflammatory response, partly characterized by edema and subsequent swelling of the muscle tissue [25]. Indices of soreness typically rise in concert with the swelling response [25]. In the present study muscle soreness increased above baseline levels for all groups at all time points. However, the supplement or timing of ingestion had no effect on soreness scores. These results disagreed with Shimomura et al. [11] who found that 5 grams of BCAAs administered prior to resistance training lowered muscular soreness in both male and female participants relative to a placebo condition. The present study also administered a protein bolus containing approximately 5 grams of BCAAs, indicating that the discrepancy may be related to the form in which the amino acids were delivered (whole protein vs. amino acid supplement).

### Possible practical and research implications

The discrepancies found in the present study compared to studies which found positive results with intact proteins and endurance exercise [1], or free form amino acids and resistance training [18] make it difficult to provide solid practical applications for sports nutritionists. To date, it appears that during endurance events that athletes can benefit from intact proteins, while during resistance training studies there appears to be consistent evidence that free form amino acids lower indices of muscle damage. However, in the present study, and in the study by Wojcik et al. [3], intact proteins do not have significant effects on indices of muscle damage when administered immediately before or following eccentric exercise. Similarly, Tipton et al. [26] found no differences between pre and post exercise whole protein ingestion on protein balance, which contrasted with past studies [27] which found greater protein balance when free form amino acids were ingested prior to rather than post exercise. While we were analyzing muscle tissue damage rather than protein balance, it is possible that in our study and in the results of Tipton et al. that whole proteins administered prior to exercise do not allow for enough time for the amino acids to interact with and exert their effects on protein balance or muscle tissue damage. Future research should examine the effects of delaying ingestion of intact proteins as compared to free form amino acid mixtures on markers of muscle damage.

## Conclusion

In conclusion, maximal eccentric repetitions of the quadriceps caused increases in indices of muscle damage, loss of isometric MVC, and increases in VAS scores of muscle soreness. A carbohydrate/protein supplement given either before or following the eccentric exercise bout had no effect on any of these measured variables. These results conflict with studies which administered various combinations of free form amino acids, and may be indicative of differences in the effects of administering whole proteins as compared to individual amino acids.
